# Knowledge and Attitudes of Physicians Towards Evidence Based Medicine in Ilam, Iran

**DOI:** 10.5812/ircmj.7204

**Published:** 2013-09-05

**Authors:** Milad Rashidbeygi, Kourosh Sayehmiri

**Affiliations:** 1 Medical Student, Student Research Committee, Ilam University of Medical Sciences, Ilam, IR Iran; 2 Psychosocial Injuries Research Center, Ilam University of Medical Sciences, Ilam, IR Iran; 3 Social Medicine Department, Medicine faculty, Ilam University of Medical Sciences, Ilam, IR Iran

**Keywords:** Attitude, Knowledge, Evidence Based Medicine, Physician

## Abstract

**Background:**

Evidence based medicine (EBM) is very important in the process of decision making, diagnosis and treatment of patients. For years, medical schools have developed instructions for EBM to determine the attitude and knowledge of physicians towards EBM and their related educational needs.

**Materials and Methods:**

This study was a questionnaire study among physicians. One hundred twenty physicians were selected using stratified random sampling in Ilam. A main outcome measure was attitudes and knowledge of physicians toward EBM, ability to access and interpret evidence, and best method of moving from opinion based to EBM.

**Results:**

Of the 120 questionnaires we have sent, 94 (78.3%) were answered. 56.6% were using the internet to answer their patients questions and 23.8% used the internet to obtain clinical evidence. Mean and standard deviation (SD) of knowledge and attitude scores were 24% ± 23% and 72% ± 10%, respectively. Pearson correlation shows a significant relation between knowledge of physicians and years of graduation (r = -0.37, P = 0.00). There was a significant difference between mean of knowledge score of general practitioners, specialist and subspecialist (P = 0.026).

**Conclusions:**

Knowledge and attitude of young physicians were more based on EBM compare to old physicians. A significant difference in knowledge mean score of physician shows that the EBM is still new in Iran, the future physician’s critical need to learn EBM and necessity of entering EMB at all medical levels.

## 1. Background

Evidence based medicine (EBM) means correct and wise use of optimal available evidence in the process of clinical decision making for patient care. EBM is very important for an optimal diagnosis and treatment of patients. In recent years, medical schools have developed educational instruction for EBM (1). EBM combines the superlative available evidence from clinical research and clinical evidence to achieve the best outcome (2-4). With lack of awareness of relevant research, substantial part of clinical practice in health relies on practitioners’ personal experience, resulting in large variation in practice between health care workers (5, 6). Physicians are being encouraged to apply EBM to improve their clinical care worldwide(7). EBM offers a systematic and structured approach of medical education (8). It sensitizes physicians for necessity of updating advances in their practicing field (9, 10) so it helps physicians during the process of decision making for an optimal treatment. Only few Iranian physicians integrate EBM into their everyday practice; we conducted surveys among physician to determine their attitudes and knowledge about EBM.

## 2. Objectives

Only few Iranian physicians integrate EBM into their everyday practice; we conducted surveys among physician to determine their attitudes and knowledge about EBM.

## 3. Materials and Methods

### 3.1. Study Design and Sample

This study was a survay among physicians. In December 2010, after approval of University Ethics Committee we sent questionnaire forms to 120 physicians in Ilam, a city in the western part of Iran. The survey represents 60% of 200 Ilam physicians obtained from a national database, which stratified random sampling, were used for choosing physician. We define strata as: General practitioners (Gp), Specialist (Sp) and sub Specialist (sub Sp). Questionnaire consists of three main sections. The first section includes their personal and professional characteristics such as: age, total years of graduation, medical specialty and sub specialty, extra training in EBM, and number of hours per day spent in different clinical arenas. In the second section, attitudes towards EBM were determined using a table with questions. In the third section, two tables with questions were designed to evaluate their knowledge about the potential resource of EBM; and their understanding of technical terms (Appendix 1). Questionnaire was used knowledge Cronbach’s Alpha coefficient assess reliability of questioner. It estimated 93% and 92% for knowledge and attitude, respectively.

### 3.2. Statistical Analysis

Results were expressed as mean ± SD. Association between variables knowledge, attitude, age, number of hours spent in clinical arenas and years of graduation were tested using Spearman’s rank correlation coefficients and Pearson correlation coefficients. Kolmogrov-Smirnov test was used to test the normality of continues variables. When normal distribution assumption was not met for some variables, the square root transformation was used to change continues variables into normal distribution. The attitude score was calculated with mean of defined score as follows:

We determined three options for each question, and three score to (Agree) option, two score to (No opinion) option and one score to (Disagree) option. 

Factor analysis's show that the knowledge of physicians toward EBM consists of two main compartments:

1-Source of EBM

2-Technical term of EBM

The knowledge score was calculated with the sum mean of defined score of two-compartment as follows:

Four options were determined for each question of source of EBM table, and four score to (Used to help in clinical decision making) options, three score to (Read) option, two score to (A ware but not used) option and one score to (Unaware) option. We determined four options for each question of Technical term in EBM table, and four score to (Understand and could Explain to others) option, three score to (Some understanding) option, two score to (Don’t understand but would like to) option and one score to (It would not be helpful for me to understand) option. To compare knowledge and attitude of physician toward EBM according to sex and physician grade, two ways ANOVA was used. Statistical analyses were done using R software. P-value less than 0.05 were considered significant.

## 4. Results

Of the 120 questionnaires, we received 94 questionnaires (78.3%). Table 1 shows the demographic features of responders. Only 12% of physicians passed certain EBM workshop. 56.6% have used the internet to answer the questions about their patients, 23.8% have used it as first priority reference to obtain clinical evidence and 61.4% have used Medline to find clinical evidence. Only 38.5% of respondents had access to internet at their department, while 3.6% had access at their local library and 57.9% at their home. 

**Table 1.** Demographic Characteristics of Physicians

Characteristics	Parameter	Value
**Gp**	26	27.7
**Sp**	62	66
**Sub Sp**	6	6.4
**Sex**		
Men		65 (69.1)
Women		29 (30.9%)
**Age**	Mean ± SD	38.8 ± 5.5
**Number of hours per day spent in clinical arenas**	Mean ± SD	8.3 ± 2.1
**Total years of graduation**	Mean ± SD	7 ± 5.3

92.6% of physicians believed that practicing EBM improves patients’ management, and 42.5% believed that doesn't barrier in the use of EBM (Table 2). Only 5.3% of physician used Cochrane Database of Systematic Reviews (Part of Cochrane), 8.5% used Evidence based medicine (BMJ publishing group) in order to support the process of clinical decision making (Table 3), and 33% could explain relative Risk to other persons (Table 4). 

**Table 2.** Physicians Attitudes Toward EBM (Values are numbers (percentages) of Subjects Who Ticket Each Response)

Subject	Agree	No opinion	Disagree
**Practicing EBM improves patients’ management.**	87/94 (92.6)	6/94 (6.4)	1/94 (1.1)
**EBM is lead to lower costs of the health system.**	78/94 (83)	15/94 (16)	1/94 (1.1)
**Diagnosis is essential reliable information.**	86/94 (91.5)	7/94 (7.4)	1/94 (1.1)
**EBM is essential learning for all physicians.**	69/94 (73.4)	16/94 (17)	9/94 (9.6)
**Doesn't barrier in use the EBM.**	40/94 (42.5)	26/94 (27.7)	28/94 (29.8)

**Table 3.** Awareness and Perceived Usefulness of Relevant Information Source (Values are Numbers (Percentages) of Subjects who Ticket Each Response)

Publication	Unaware	A ware but not used	Read	Used to help in clinical decision making
**Bandolier (published in Oxford)**	76/93 (81.7)	10/93 (10.8)	5/93 (5.3)	2/93 (2.2)
**EBM (BMJ publishing group)**	61/94 (64.9)	15/94 (16)	10/94 (10.6)	8/94 (8.5)
**Effective Health Care Bulletins (Universities of Leeds and York)**	71/92 (77.2)	10/92 (10.9)	8/92 (8.7)	3/92 (3.3)
**Cochrane Database of systematic Reviews (Part of Cochrane)**	75/94 (79.8)	6/94 (6.4)	8/94 (8.5)	5/94 (5.3)
**DARE**	78/92 (84.8)	11/92 (12)	3/92 (3.3)	0/92 (0.0)
**Evidence Based Purchasing (South and West R&D)**	79/92 (85.9)	7/92 (7.6)	5/92 (5.4)	1/92 (1.1)

**Table 4.** Understanding of Technical Term used in EBM (Values are Numbers (Percentages) of Subject who Ticket Each Response)

Term	It would not be helpful for me to understand	Don’t understand but would like to	Some understanding	Understand and could Explain to others
**Relative risk**	13/94 (13.8)	23/94 (24.5)	27/94 (28.7)	31/94 (33)
**Absolute risk**	12/94 (12.8)	27/94 (28.7)	34/94 (36.2)	21/94 (22.3)
**Systematic review**	14/94 (14.9)	31/94 (33)	27/94 (28.7)	22/94 (23.4)
**Odds ratio**	15/93 (16.1)	30/93 (32.3)	28/93 (30.1)	20/93 (21.5)
**Meta-analysis**	16/94 (17)	38/94 (40.4)	21/94 (22.3)	19/94 (20.2)
**Clinical effectiveness**	12/94 (12.8)	32/94 (34)	29/94 (30.9)	21/94 (22.3)
**Confidence interval**	14/94 (14.9)	39/94 (41.5)	25/94 (26.6)	16/94 (17)
**Publication bias**	12/94 (12.8)	28/94 (29.8)	32/94 (34)	22/94 (23.4)

**Figure 1.**
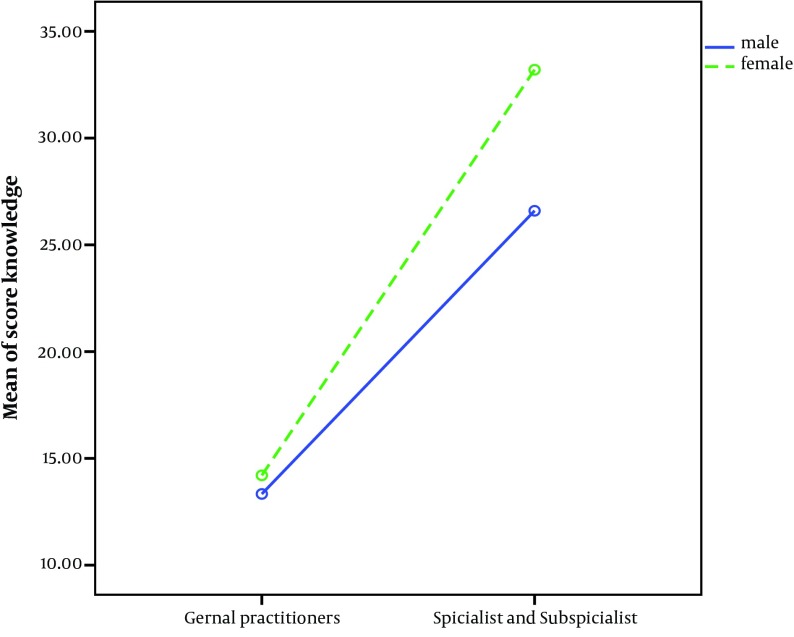
Comparisons Mean of Physicians Knowledge Score According to Sex

Mean and SD knowledge score and attitude score were 24% ± 23%, 72% ± 10% respectively. A two way ANOVA show that knowledge score of physicians female more than physicians male (P = 0.01) (Figure 1). 

Twelve present of respondents said that “I had passed an EBM course”. There was no statistical significant difference between mean score of knowledge about EBM in those who passed an EBM workshop and those who did not pass this workshop (P = 0.42). Pearson correlation showed a significant association between knowledge of physicians and years of graduation (r = -0.37, P = 0.00). Negative correlation was seen between years of graduation, and the score of knowledge (Table 5). 

**Table 5.** Pearson Correlation Coefficient of Knowledge, Attitude, Age, Years of Graduation and Number of Hours per Spent in Clinical Arenas

		Knowledge	Attitude	Age	Number of hours per spent in clinical arenas	Years of graduation
**Knowledge**	r	1	0.15	-0.13	0.05	-0.36
	P		0.13	0.22	0.59	0.0
**Attitude**	r		1	-0.05	0.04	-0.11
	P			0.60	0.65	0.28
**Age**	r			1	-0.40	0.65
	P				0.00	0.00
**Number of hours per spent in clinical arenas**	r				1	-0.30
						0.00
**Years of graduation**	r					1
	P					

There was a significant difference between mean of knowledge score of general practitioners, specialist and subspecialist (P = 0.026), whereas physicians attitude toward EBM were not significantly different (P = 0.73). 57.8% of physicians have believed that partial EBM is used in Iran.

## 5. Discussion

This study was the first study to consider attitude and knowledge of graduate physicians in Iran and first study to compare EBM among general practitioner,, specialist and sub specialist physicians.

Response rates of 78.3% an important achievement as the response rate to the survey questionnaire among physicians, more than in the study of McAvoy (11). The difference between respondents’ attitudes and their understanding of respondents show more positive attitude toward EBM than non-respondents (12). 57.8% of physicians have believed that partial EBM was used in Iran, this estimation is below the previous studies, which found that more than 80% of the activity in general practice is evidence based (13). However, our finding from self-made question is limited to this study.

Used methods were criticized because based on previous study, the quality of evidence was not reviewed and non-experimental evidence was included (14). Respondents showed a low level of awareness of extracting medical journals, review update medical publications, and databases about EBM. Effort have been made to find out who uses the Cochrane Source (15) and whether gynecologists were informed of and used it (16), but there have been no such studies to compare physicians. The practice of EBM involves use of best available evidence in clinical decision making for patient care from systematic investigations (17). Much of this clinical evidence has already been identified, critically evaluated, and collected in journals and databases (18).

About medical education resources such as Cochrane and Dare, most physicians do not know this resource and only 10.6% did use it; this amount was less in the previous study (17). Our respondents showed a low knowledge of the technical terms used in EBM. Therefore, emphasis on training clinical epidemiological to physicians for use data such as Relative Risk and Odds Ratio in Clinical decision is important.

12% of respondents said that “I had passed an EBM course”. There was no statistical significant difference between mean score of EBM knowledge in those passed the workshop and those do not passed the workshop (P = 0.42), this estimate showed EBM have been institutionalized in doctors practice.

Pearson correlation showed significant different between mean of knowledge score of general practitioners, specialist and subspecialist (P= 0.026), which confirms EBM should be entered at all medical levels in Iran.

Significant association between knowledge of physicians and years of graduation(r = -0.37, P = 0.00) estimated that young physicians’ knowledge and attitude were more than old physician.

EBM is considered to be important for diagnosis and treatment of diseases. According to low awareness of Ilam physicians about EBM and their interest to learn it, introduction and learning course of EBM for graduated general practitioners and specialists seems more necessary. Young physicians’ knowledge and attitude is higher than older physician and a significant difference in knowledge mean score of physician exist; this shows that the EBM is still new for Iranian physicians and the future physician’s critical need which should be entered at all medical levels.

## Appendix 1

**Part 1: Personal data**

- Age: …….

-Sex: male O female O

-General Practice O Specialist O sub Specialist O

- Number of hours per spent in clinical arenas: …….

- Years of graduation: …….

-Do you have Internet access? Yes O No O

- In which of the following locations have access to the Internet?

Home O Clinic O Library O Other O

- If you use the Internet for clinical evidence, what sites do you use?

Medline O Specific sites O Other O

…………………………………………………….

**Part 2: Knowledge to EBM**

**Table A1.** Source of EBM

Publication	Unaware	A ware but not used	Read	Used to help in clinical decision making
**Bandolier (published in Oxford)**				
**Evidence based medicine (BMJ publishing group)**				
**Effective Health Care Bulletins (Universities of Leeds and York)**				
**Cochrane Database of systematic Reviews (Part of Cochrane)**				
**DARE**				
**Evidence Based Purchasing (South and West R&D)**				

**Table A2.** Technical term of EBM

Term	It would not be helpful for me to understand	Don’t understand but would like to	Some understanding	Understand and could Explain to others
**Relative risk**				
**Absolute risk**				
**Systematic review**				
**Odds ratio**				
**Meta-analysis**				
**Clinical effectiveness**				
**Confidence interval**				
**Publication bias**				

**Table A3.** Attitude to EBM

Subject	Agree	No opinion	Disagree
**Practicing evidence based medicine improve patients management.**			
**Evidence based medicine is lead to lower costs of the health system.**			
**Diagnosis is essential reliable information.**			
**Evidence based medicine is essential learning for all physicians.**			
**Barrier doesn't exist in use the evidence based medicine.**			

- In your opinion, how much of our current practice in Iran is based on evidence?

Much O Medium O Low O
